# Cocaine and COVID-19 in ST-Elevation Myocardial Infarction

**DOI:** 10.1155/2022/5640965

**Published:** 2022-04-22

**Authors:** Florian Appenzeller, Meinrad Gawaz, Iris Müller

**Affiliations:** University Hospital, Department of Cardiology and Cardiovascular Medicine, Eberhard Karls University Tübingen, 72076 Tübingen, Germany

## Abstract

Both COVID-19 disease and cocaine consumption have prothrombotic and hypercoagulable effects and are associated with increased risk of cardiovascular events. We report the case of a patient with acute myocardial infarction in the setting of active COVID-19 disease and recent cocaine consumption. We hypothesize that COVID-19 and cocaine synergistically provoke cardiovascular events. Identifying COVID-19 disease and/or cocaine abuse as potential triggers of acute myocardial infarction can be crucial due to distinctive therapeutic consequences.

## 1. Introduction

Cocaine promotes acute myocardial ischemia and infarction by exerting various adverse effects on myocardium and coronary arteries: coronary vasoconstriction (increased levels of endothelin-1, impaired NO production, and alpha receptor stimulation), shift to a prothrombotic state (increased platelet activity and aggregation, elevated levels of fibrinogen, and von Willebrand factor), and increased myocardial oxygen demand (elevated heart rate, blood pressure, and myocardial contractility). The increase in risk for myocardial infarction after cocaine consumption has a volatile character with prompt onset and rapid decrease in the first two hours thereafter [1–6].

Myocardial perfusion impairment in COVID-19 underlies complex pathophysiological mechanisms including direct viral cellular toxicity and systemic hyperinflammation with cytokine-mediated damage leading to endothelial dysfunction, platelet hyperactivation, hypercoagulability, and atherosclerotic plaque destabilization predisposing towards micro- and macrovascular thrombotic occlusion, potentially resulting in myocardial infarction [7–9].

## 2. Case Presentation

**Figure 1:**
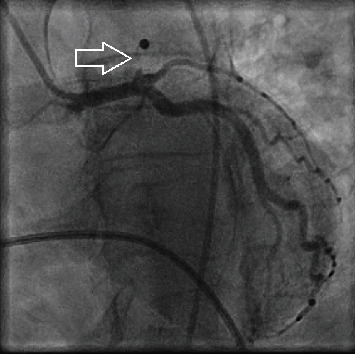
Angiography of the left coronary arteries revealing total occlusion of proximal LAD (arrow).

**Figure 2:**
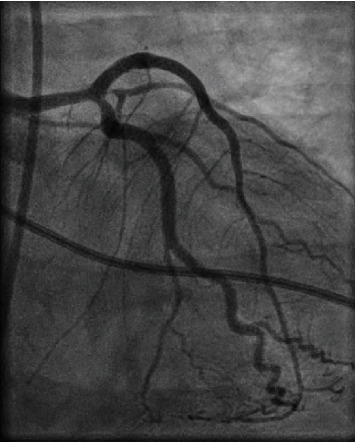
Restored perfusion established by percutaneous coronary angioplasty and stent implantation.

A 38-year-old male presented to the emergency department with severe pain and tightness in his left chest without radiating into adjacent body parts. The symptoms had an acute onset approximately 45 minutes earlier while the patient was at rest. Upon arrival, we were informed about the patient's current COVID-19 infection diagnosed five days prior with previously mild course consisting of fever, shortness of breath, and fatigue. In addition, the patient conceded intranasal consumption of cocaine approximately one hour before onset of symptoms. Besides habitual nicotine and cocaine consumption, further cardiovascular risk factors were negated and no similar events have occurred to date. Medical history excluded other significant preconditions. Electrocardiogram showed ST-segment elevations in anterior and lateral leads implicating transmural myocardial infarction. Subsequently, intravenous acetylsalicylic acid and heparin were administered, and transportation to our catheterization laboratory was initiated. On the way, one episode of ventricular fibrillation occurred followed by cardiopulmonary resuscitation and external defibrillation with immediate return of spontaneous circulation. The patient arrived with spontaneous breathing and sufficient circulatory parameters. Coronary angiography was performed revealing total occlusion of the proximal left anterior descending (LAD) coronary artery in the setting of minor general coronary sclerosis (Figure 1). Coronary angioplasty and drug-eluting stent implantation fully restored perfusion of the LAD (Figure 2). Antithrombotic regime with acetylsalicylic acid was continued, and prasugrel was added. Initial echocardiography depicted extensive hypokinetic wall motion abnormality consistent with the culprit lesion. Laboratory results showed an initial troponin-I of 21,766 ng/l (<37 ng/l) and a peak in total creatine kinase at 3,065 U/l (<170 U/l) as well as elevated D-dimers of 2.1 *μ*g/ml (<0.5 *μ*g/ml) and IL-6 of 72 ng/l (<4 ng/l). COVID-19 infection was confirmed via reverse transcriptase-polymerase chain reaction. Consequently, the patient was isolated in our intensive care unit and received argatroban, remdesivir, and dexamethasone in accordance with current COVID-19 guidelines. There were no clinical signs of acute respiratory distress with a normal respiratory rate at rest and oxygen saturation on room air around 95%. Chest X-ray ruled out suspicious pulmonary opacities. Ultimately, former wall-motion abnormalities were regressive with residual minor systolic left ventricular impairment. The patient showed adequate clinical improvement and was discharged six days after admission. GRACE-score predicted a 6-month postdischarge mortality of 4%.

## 3. Discussion

Chest pain is the leading cause of cocaine-associated emergency department visits [10]. Acute coronary syndrome (ACS) associated with cocaine abuse differs from cocaine-unrelated ACS: myocardial infarction is considerably less likely to be confirmed [11], and suspected ST-elevation myocardial infarction (STEMI) is more likely to be revealed as false STEMI (i.e., nonocclusive myocardial infarction) [12]. Furthermore, these patients are more likely to be of younger age and male gender and to have lower socioeconomic status, while common cardiovascular risk factors such as diabetes and hypertension are less frequent [13].

COVID-19-associated ACS has been frequently described; however, data related to occlusive myocardial infarction are limited. Without differentiating between STEMI and non-STEMI, a register-based study estimated the incidence of acute myocardial infarction to be around five times higher in the first two weeks after COVID-19 diagnosis compared to a previous intraindividual control interval [14].

Here we describe the first case to our knowledge of STEMI in the unusual setting of both acute COVID-19 disease and prior cocaine consumption. Since both conditions exert various cardiovascular effects and are associated with thrombotic complications, a synergistic effect towards coronary events seems plausible. It remains speculative what proportion of cause is attributed to cocaine and/or COVID-19 in this case.

## 4. Conclusions

While laboratory screening for COVID-19 in every patient referred to our hospital is common practice, there is no routine assessment for illegal drug abuse in patients presenting with ACS. This is partially explained by the relatively low prevalence of cocaine consumption in our catchment area. Assessing illegal drug abuse by questioning and/or toxicological screening should be considered particularly in younger patients with a low to moderate cardiovascular risk profile presenting with ACS.

The underlying cause has neglectable consequence on guideline-recommended primary percutaneous coronary intervention and antiplatelet strategy in STEMI but there are therapeutic implications that should be considered, respectively: beta-blockers may aggravate cocaine-induced coronary spasm and should be avoided in the acute event while intravenous nitroglycerin and benzodiazepines were validated beneficial in this scenario [15]. In COVID-19 disease and STEMI, anticoagulation in addition to antiplatelet therapy should be carefully assessed in order to prevent thromboembolic complications depending on individual bleeding and thrombotic risk [16]. Current guidelines should be considered in order to do justice to the rapidly evolving pharmacological strategies in COVID-19 disease.
